# Hypothyroidism Intensifies Both Canonic and the *De Novo* Pathway of Peroxisomal Biogenesis in Rat Brown Adipocytes in a Time-Dependent Manner

**DOI:** 10.3390/cells10092248

**Published:** 2021-08-30

**Authors:** Marija Aleksic, Igor Golic, Andjelika Kalezic, Aleksandra Jankovic, Bato Korac, Aleksandra Korac

**Affiliations:** 1 Center for Electron Microscopy, Faculty of Biology, University of Belgrade, 11000 Belgrade, Serbia; marija.aleksic@bio.bg.ac.rs (M.A.); igor.golic@bio.bg.ac.rs (I.G.); or b.korac@bio.bg.ac.rs (B.K.); 2 Department of Physiology, Institute for Biological Research “Sinisa Stankovic”—National Institute of Republic of Serbia, University of Belgrade, 11000 Belgrade, Serbia; andjelika.kalezic@ibiss.bg.ac.rs (A.K.); aleksandra.jankovic@ibiss.bg.ac.rs (A.J.)

**Keywords:** hypothyroidism, brown adipocyte, peroxisome, biogenesis pathway, inter-organellar association

## Abstract

Despite peroxisomes being important partners of mitochondria by carrying out fatty acid oxidation in brown adipocytes, no clear evidence concerning peroxisome origin and way(s) of biogenesis exists. Herein we used methimazole-induced hypothyroidism for 7, 15, and 21 days to study peroxisomal remodeling and origin in rat brown adipocytes. We found that peroxisomes originated via both canonic, and *de novo* pathways. Each pathway operates in euthyroid control and over the course of hypothyroidism, in a time-dependent manner. Hypothyroidism increased the peroxisomal number by 1.8-, 3.6- and 5.8-fold on days 7, 15, and 21. Peroxisomal presence, their distribution, and their degree of maturation were heterogeneous in brown adipocytes in a Harlequin-like manner, reflecting differences in their origin. The canonic pathway, through numerous dumbbell-like and “pearls on strings” structures, supported by high levels of Pex11β and Drp1, prevailed on day 7. The *de novo* pathway of peroxisomal biogenesis started on day 15 and became dominant by day 21. The transition of peroxisomal biogenesis from canonic to the *de novo* pathway was driven by increased levels of Pex19, PMP70, Pex5S, and Pex26 and characterized by numerous tubular structures. Furthermore, specific peroxisomal origin from mitochondria, regardless of thyroid status, indicates their mutual regulation in rat brown adipocytes.

## 1. Introduction

Peroxisomes are highly dynamic organelles able to rapidly adapt to cellular metabolic requirements by changing their abundance, shape, size, and enzyme profile within [1]. Peroxisomes perform various functions that differ depending on the species, organism, cell type, and environmental conditions. In mammalian cells, peroxisomes are involved in lipid metabolism and their main function is β-oxidation of fatty acids [2].

Peroxisomal biogenesis has been well studied but gaps in our knowledge remain. The formation of peroxisomes from pre-existing ones by “growth and division” is common and is the canonic pathway for biogenesis [3,4]. *De novo* peroxisomal biogenesis is still ill-defined [5]. Peroxisomes can originate *de novo* by “budding” from the smooth endoplasmic reticulum (sER). However, recent studies indicated transport and communication between mitochondria and peroxisomes by mitochondria-derived vesicles [6]. Based on previous evidence, Mohanty and McBride proposed that both ER and mitochondria-derived vesicles could contribute to peroxisomal biogenesis [7]. At last, Sugiura et al. (2017) provided evidence for the hybrid nature of newly produced peroxisomes within human fibroblasts from patients with Zellweger syndrome normally lacking peroxisomes [8]. Whether peroxisomes can arise *de novo* from mitochondria in physiological or other pathological conditions and in vivo remains an open question.

The main molecular players that drive peroxisomal biogenesis are peroxisomal membrane proteins (membrane transporter PMP70) and peroxins (Pex), that participate in peroxisomal division (Pex11β), *de novo* peroxisomal biogenesis (Pex16 and Pex19), and peroxisomal structural/functional maturation (Pex16, Pex19, Pex5, Pex13, Pex26, and Pex6). Pex expression and general control of peroxisomal proliferation are both under transcriptional control of the peroxisome proliferator-activated receptor (PPAR) family of transcriptional factors—PPARα and PPARγ [9].

Brown adipose tissue (BAT) is a highly specialized thermogenic organ that uses lipids stored in lipid bodies as the main substrate for oxidation in mitochondria to produce heat. Data regarding peroxisomal presence and function in BAT are limited. In brown adipocytes, peroxisomes were first identified at the ultrastructural level by Ahlabo and Barnard (1971) and further studied by Pavelka et al. (1976) [10,11]. All other studies predominantly focused on their role in lipid metabolism and cooperation with mitochondria in β-oxidation of fatty acids during thermogenesis, probably due to their scarcity compared to mitochondria and lipid bodies. Therefore, as has been demonstrated, peroxisomal proliferation in response to cold exposure supports thermogenesis and increases β-oxidation enzyme activity, along with the level and activity of catalase [10,12,13]. Despite the extremely important role of peroxisomes in BAT metabolism [14], especially in lipid metabolism in thermogenically activated BAT, peroxisomal origin and pathway(s) of peroxisomal biogenesis remained unexplored.

Bearing all this in mind, our current study aimed to address the peroxisomal biogenesis pathways in brown adipocytes using hypothyroidism as an established experimental model for the induction of peroxisomal proliferation [15]. To induce hypothyroidism, rats were treated with the antithyroid drug methimazole for either 7, 15, or 21 days. A specific cytochemical DAB staining technique and immunogold labelling of catalase (CAT), as a peroxisomal marker, were used to identify peroxisomes and analyze peroxisomal proliferation and maturation in rat brown adipocytes over the time course of hypothyroidism. Expression of peroxins involved in both canonic and *de novo* pathways of peroxisomal formation and maturation and the level of PPARα and PPARγ as main transcriptional regulators of peroxisomal biogenesis were also scrutinized.

## 2. Materials and Methods

### 2.1. Animals and Experimental Design

All procedures performed in this experiment were approved by the Ethics committee for the treatment of experimental animals of the Faculty of Biology at the University of Belgrade and by the Veterinary Directorate of the Ministry of agriculture and environmental protection of the Republic of Serbia (ethical approval code: 323-07-07505/20l5-05/4). Two-month-old Wistar rats (330 ± 30 g) were maintained under 22 ± 1 °C and 12 h light/dark cycles with ad libitum access to standard pelleted food. Animals were divided into four groups, each consisting of eight animals. Three groups were treated with 0.04% methimazole (Methimazole crystalline M8506, Sigma-Aldrich, Steinheim, Germany) in drinking water for 7, 15, and 21 days, respectively; animals in the fourth group served as control—the euthyroid group (drinking tap water). After twenty-one days, animals were sacrificed using a decapitator (Harvard Apparatus, Holliston, MA, USA). To assess the effectiveness of the methimazole treatment, T3, T4, and TSH serum levels were analyzed (Supplementary Materials 1).

### 2.2. Transmission Electron Microscopy

Small parts of BAT immediately after dissection were fixed with 2% glutaraldehyde/2% paraformaldehyde in 0.1 M Sørensen phosphate buffer (PB, pH 7.2) for 1 h at 4 °C. After fixation, tissue was rinsed in phosphate buffer and then preincubated in 0.1% 3,3′-diaminobenzidine (DAB) (Sigma-Aldrich, Steinheim, Germany) in 0.1 M Sǿrensen phosphate buffer for 30 min, for peroxisomal staining [16]. Furthermore, in the preincubation medium, 0.01% H_2_O_2_ was added and incubated for 1 h at 37 °C. After washing in phosphate buffer, tissue was postfixed in 2% osmium tetroxide in the same buffer, then routinely dehydrated using increasing concentrations of ethanol and embedded in Araldite (Fluka, Buchs, Switzerland). Ultra-thin sections of BAT were obtained using a Leica UC6 ultramicrotome (Leica Microsystems, Wetzlar, Germany) and mounted on copper grids. Sections were examined on a Philips CM12 transmission electron microscope (Philips/FEI, Eindhoven, The Netherlands) equipped with the digital camera SIS MegaView III (Olympus Soft Imaging Solutions, Münster, Germany). The obtained electron micrographs were used for morphological/stereological analyses of BAT.

### 2.3. Stereological and Morphometric Analyses

Forty nucleated brown adipocyte profiles were analyzed per group. The relative peroxisomal number was calculated using the equation: N = (Ntotal/Pcell)* 100 µm^2^, where Ntotal/Pcell is the total number of peroxisomes per cell divided by the total area of the cell, and relative to 100 µm^2^ of the cell surface. The volume density of peroxisomes was calculated using a derivation of the Deless equation: Vv = Po/Ptotal, where Po/Ptotal is the point fraction of the total points hitting the organelle of interest divided by the total points hitting the adipocyte [17]. All these measurements and analyses were performed using ImageJ software (NIH, Bethesda, MD, USA).

### 2.4. Immunofluorescence

Semi-thin sections of interscapular BAT were used for standard immunolabeling procedure, using a primary antibody against CAT and an appropriate fluorochrome-conjugated secondary antibody (1:400; Alexa Fluor^®^ 488 goat anti-rabbit, Thermo Fisher Scientific, Waltham, MA, USA). Sytox orange (1 μL ml^−1^, Thermo Fisher Scientific, Waltham, MA, USA) was used for nuclei counterstaining. Slides were mounted with Mowiol (Polysciences, Eppelheim, Germany), and confocal images were acquired with a Leica TSC SP8 confocal microscope (Leica Microsystems, Wetzlar, Germany) using 63/1.4 NA oil immersion lens. The specificity of immunofluorescence was tested by the omission of the primary antibody.

### 2.5. Immunogold

Ultra-thin sections of BAT were obtained using a Leica UC6 ultramicrotome (Leica Microsystems, Wetzlar, Germany) and mounted on nickel grids. After antigen retrieval in 10 mM citrate buffer and incubation with 5% BSA in Tris-buffered saline/0.1% Tween 20 (TBS-T) for 1 h at room temperature, grids were incubated overnight at 4 °C with the primary anti-CAT antibody (1:500, ab1877) or 1 h at 37 °C with the anti-voltage-dependent anion channel 1 (VDAC1) antibody (1:150, ab34726) and anti-Drp1 antibody (ab93942). After rinsing in TBS-T, grids were incubated with the 10-nm gold-conjugated anti-rabbit secondary antibody for CAT and Drp1 (1:20, ab27234) or the 20-nm gold-conjugated anti-rabbit secondary antibody for VADC1 (1:20; ab27237), for 1 h at room temperature, rinsed in TBS-T and double distilled water, air-dried, and examined with a Philips CM12 transmission electron microscope (Philips/FEI, Eindhoven, The Netherlands). The specificity of the immune reactions was tested by omitting the primary antibody.

### 2.6. Western Blotting

After decapitation, the right portion of interscapular BAT was frozen immediately. Later, the protein content was estimated by the method of Lowry et al. [18]. Primary antibodies against Pex11β (1:1000, ab74507), Pex19 (1:2000, ab137072), Pex16 (1:500, sc-398189), Pex5 (1:1000, sc-137103), Pex13 (1:1000, sc-271477), Pex26 (1:500, sc-376817), Pex6 (1:500, sc-271813), PMP70 (1:1000, ab74507), catalase (1:800, ab1877), calnexin (1:1000, ab22595), dynamin-related protein 1—Drp1 (1:1000, ab93942), PPARα (1:2000, ab8934), and PPARγ (1:400, ab19481) were purchased from Abcam (Abcam, Cambridge, UK) or Santa Cruz (Santa Cruz Biotechnology, Dallas, TX, USA). All used antibodies were chosen as they recognize the exact epitope, a small amino acid sequence, which increases their specificity toward the target protein. Immunoreactive bands were quantified using ImageJ software (NIH, Bethesda, MD, USA). The volume represents the sum of all pixel intensities within a band, and 1 pixel = 0.007744 mm^2^. We averaged the ratio of pixels per band for the target protein in the corresponding samples from three similar independent experiments. The mean values obtained from the euthyroid group were taken as 100%, and those from methimazole-treated groups were expressed as percentages against the euthyroid group.

### 2.7. Statistics

All data obtained were analyzed by the software GraphPad Prism (GraphPad Prism, Version 5.03). Normal distribution was tested by D’Agustino and Pearson. If normality criteria were met, one-way ANOVA with posthoc multiple comparison test was run; if normality criteria were not met, the Kruskal–Wallis non-parametric test was run. Results are expressed as mean ± SEM of obtained values, and significances were set at *p* < 0.05.

## 3. Results

To identify peroxisomes and analyze peroxisomal biogenesis in rat brown adipocytes over the time course of hypothyroidism, we used immunofluorescent labelling of catalase and two specific methods for their visualization at the ultrastructural level, a selective cytochemical DAB technique and immunogold labelling of catalase as a peroxisomal marker.

### 3.1. Hypothyroidism Induces Peroxisomal Proliferation

**Figure 1:**
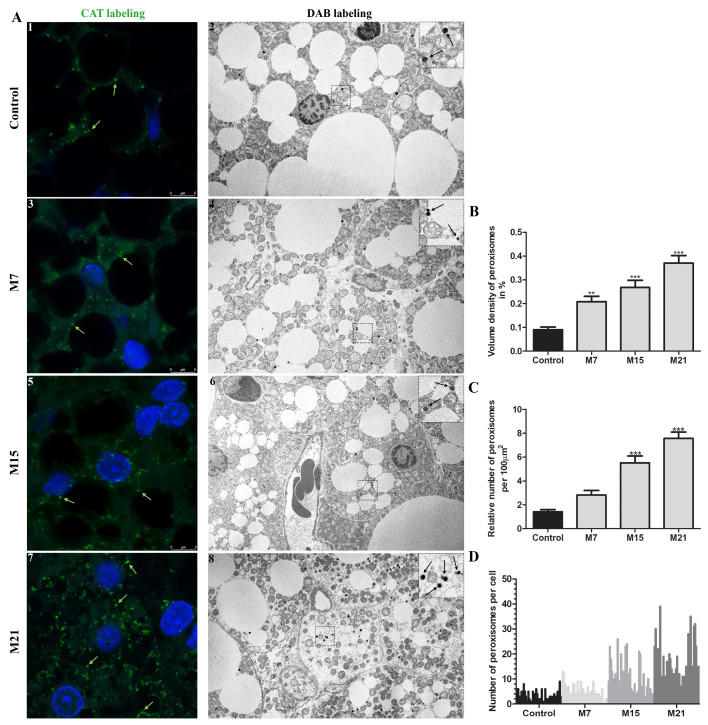
Hypothyroidism-induced peroxisomal proliferation in brown adipocytes. (**A**) Immunofluorescent (1,3,5,7), and DAB labelling (2,4,6,8) of peroxisomes (arrows) in brown adipocytes in euthyroid (Control; 1–2) and hypothyroid groups treated with methimazole for 7 (M7;3,4), 15 (M15;5,6), and 21 (M21;7,8) days, respectively. Immunofluorescent labelling of CAT (green, arrows) showed an increased presence of peroxisomes during the treatment. DAB labelling (black, arrows) indicated their number and distribution per cell in more detail. (**B**) The volume density of peroxisomes per group is presented as a percentage. (**C**) Relative number of peroxisomes per group, standardized to 100 μm^2^ of cell area, and (**D**) distribution of their number per cell within the group. Scale bars: Confocal microscopy 5 μm, electron microscopy 1 μm. Bars represent the mean ± SEM. * Compared to control, ** *p* < 0.01, *** *p* < 0.001.

In brown adipocytes of euthyroid rats, a small pre-existing quantity of peroxisomes existed, representing less than 0.1% of the cell volume. Hypothyroidism led to marked peroxisomal proliferation (Figure 1A–C). Over the time course of hypothyroidism, at each examined time point (7, 15, and 21 days), peroxisomal volume density increased significantly (Figure 1B). This was achieved exclusively by an increase in the peroxisomal number (Figure 1A,C), 1.8-, 3.6-, and 5.8-fold increase on days 7, 15, and 21, respectively, since peroxisomal diameter and shape remained unchanged, ranging from 0.1–0.3 µm (Figure 1A). Immunofluorescent labelling of CAT confirmed the existence of both globular and tubular forms. On days 15 and 21, tubular peroxisomal forms prevailed (Figure 1A; Supplementary Materials 2—video 1–4). Interestingly, the peroxisomal number varied greatly within cells leading to unequal peroxisomal distribution, e.g., a Harlequin pattern within neighboring brown adipocytes (Figure 1A,D). This heterogeneity observed in the euthyroid control was maintained over their proliferation. The peroxisomes in hypothyroidism were distributed in a similar way but variation was more prominent on days 15 and 21 (Figure 1A). In all the examined groups, we also observed a few cells without peroxisomes, particularly on day 7.

### 3.2. Hypothyroidism Intensifies Both Canonic and De Novo Pathway of Peroxisomal Biogenesis in a Time-Dependent Manner

**Figure 2:**
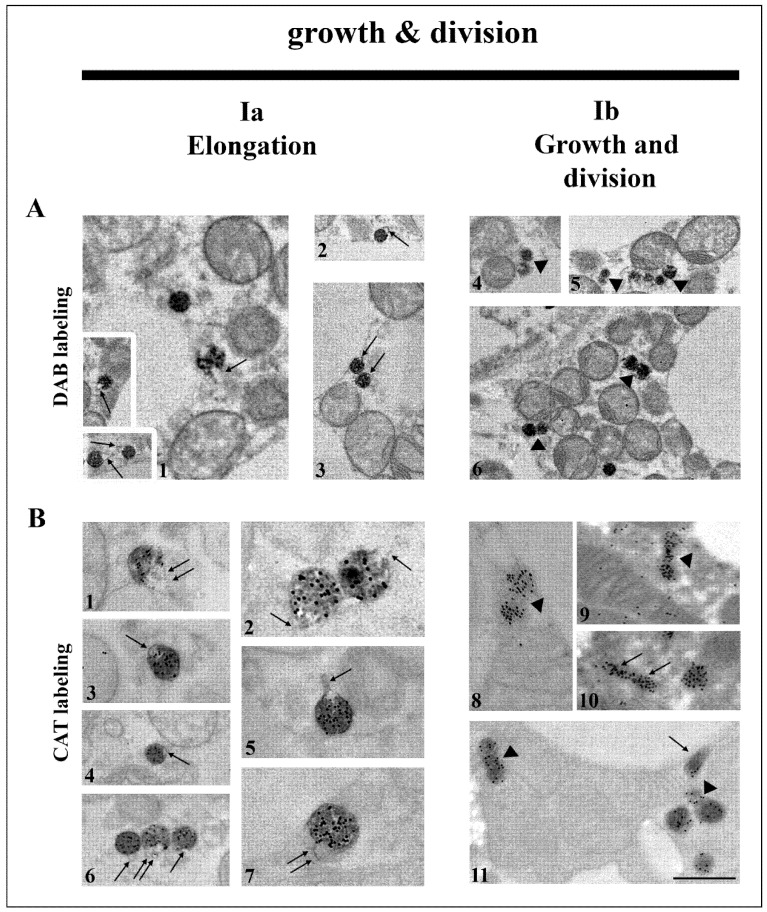
Canonic pathway of peroxisomal biogenesis through growth and division in brown adipocytes. Representative electron micrographs from both euthyroid and hypothyroid groups with DAB-labelled (**A**) and CAT-labelled (**B**) peroxisomes. This multistep pathway of biogenesis starts with the formation of membrane protrusions at spherical peroxisomes—the peroxisomal noses (**A**1–3,**B**3,4—arrows). Further elongation of noses (**B**1,2,5,6,7—arrows) leads to the formation of tubular structures (**B**10,11—arrows), which is supported by the import of new peroxisomal membrane proteins and lipids. The following step is the peroxisomal membrane constriction (segmentation of the membrane compartment) and forming dumbbell-like structures (**A**4,5,6, and **B**8,9,11—arrowheads), which leads to final division. Original magnification 17,000× and 22,000× (**B**2,5,7). Scale bar 1 μm.

As our results indicated marked peroxisomal proliferation in brown adipocytes over the course of hypothyroidism, we further examined which of the two established pathways of biogenesis were responsible for the increase in their number. Using both DAB- and CAT-labelled peroxisomes to study their origin and maturation, we found that canonic and *de novo* pathways of peroxisomal biogenesis (Figure 2 and Figure 4) take place in both euthyroid control and over the time course of hypothyroidism.

We found that a large portion of peroxisomes formed peroxisomal tubular extensions (noses) (Figure 2A1–3,B1–7), which was followed by their elongation and constriction (division) (Figure 2A4–6,B8–11). The pronounced peroxisomal elongation was observed on day 7 of hypothyroidism, where they were the most numerous. They were characterized by a dark and homogenous DAB-labelled globular part, in contrast to DAB negative noses. Numerous dumbbell-like structures with a central narrowing part of the membrane were also observed on day 7 of hypothyroidism (Figure 2A4–6,B8,9), indicative of peroxisomal division. The newly born peroxisome was smaller than the pre-existing one and was poorly labelled by DAB (Figure 2A4–6). After 7 days of hypothyroidism, we also found a string of interconnected neighboring peroxisomes (“pearls on strings”) additionally suggesting peroxisome budding from pre-existing ones (Figure 2A5). The first peroxisome in the strings was larger and darker, while the others were smaller and poorly DAB-labelled.

**Figure 3:**
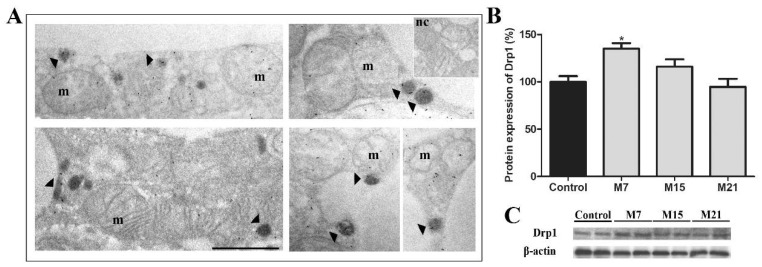
Immunogold labelling of Drp1 on representative images from the hypothyroid group treated with methimazole for 7 days (M7) (**A**) showing that the immunopositive reaction was localized on the mitochondrial outer membrane, on mitochondrial cristae, and some peroxisomes (arrowheads). m, mitochondria; dark peroxisomes labelled by DAB. Inset nc, negative control of immunolabeling. Original magnification 17,000×, scale bar 1 µm. (**B**) Protein expression of Drp1 in rat brown adipose tissue of the euthyroid control (black) and hypothyroid groups (grey) treated with methimazole for 7 (M7), 15 (M15), and 21 (M21) days, respectively. The protein content is expressed as a percentage of the control. Band images from a representative blot of three trials are shown (**C**). Bars represent the mean ± SEM. * Compared to control, * *p* < 0.05.

We further look at the extent of peroxisomal division. In addition to Pex11β, another key player in peroxisomal division in mammalian cells is Drp1. Protein expression of Drp1 significantly increased on day 7 of hypothyroidism, in comparison to euthyroid control and then returned to the control level (Figure 3B). To confirm this, we used immunogold Drp1 labelling. Our results revealed that Drp1 was localized on the mitochondrial outer membrane, on mitochondrial cristae, and on some peroxisomes (Figure 3A). Immunopresence of this division-driving protein was highest in the group treated for 7 days, which correlated with increased Drp1 protein expression.

**Figure 4:**
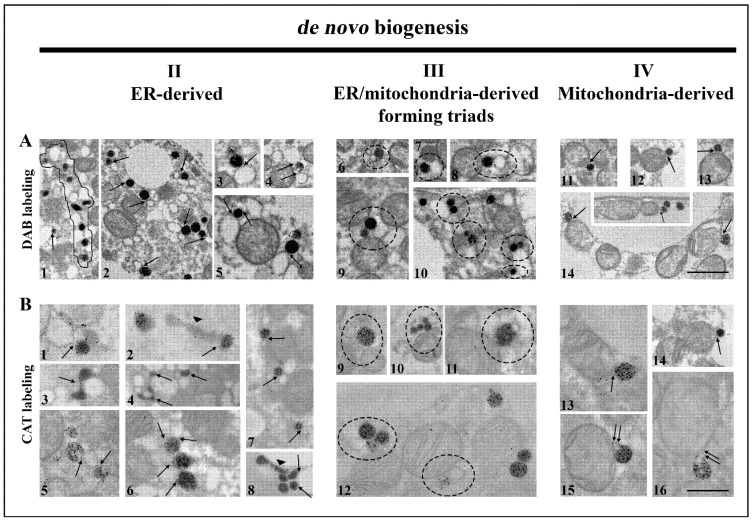
Ways of *de novo* peroxisomal biogenesis in brown adipocytes. Representative images from both euthyroid and hypothyroid groups with DAB-labelled (**A**) and CAT-labelled (**B**) peroxisomes. ER-derived peroxisomes (**A**1–5,**B**1–8) are still connected with ER cisterns (arrows), by membranous bridges that connect these two organelles (**A**2–5, and **B**1,5—arrows). Note the long pre-peroxisomal cisterns of ER (**B**2,8—arrowhead), but also structure-like “pearls on a string” (**A**1—outlined structure). ER/mitochondria-derived peroxisomes are also connected to ER and mitochondria at the same time, making triads of organelles (**A**6–10,**B**9–12—circles). Triads in brown adipocytes suggest a hybrid nature of newly born peroxisomes. Mitochondria-derived peroxisomes (**A**11–14,**B**13–16) are also connected to mitochondria via membranous bridges (arrows), suggesting that peroxisomes could originate from mitochondria in brown adipocytes. Original magnification 17,000× (**A**), and 22,000× (**B**). Scale bars 1 μm.

Frequent structural associations of peroxisomes and smooth ER (sER) tubules in both euthyroid control and hypothyroidism, which were especially numerous on day 21, were also observed. This association was formed from membranous bridges (Figure 4A1–5,B1–8), indicating peroxisomal biogenesis from the sER. The presence of many interconnected peroxisomes, such as “pearls on a string” (Figure 4A1, and Figure 8M,N), additionally supported the concept of peroxisomal biogenesis from elongated pre-peroxisomal ER cisterns.

**Figure 5:**
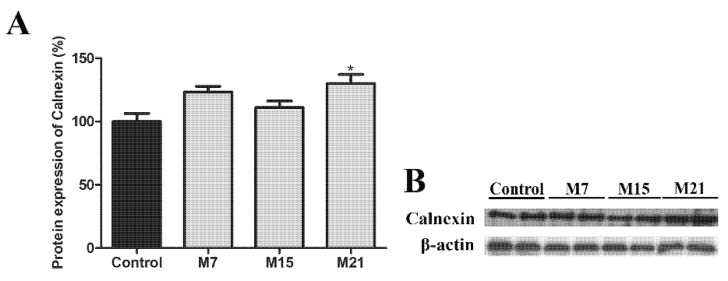
Protein expression of ER marker calnexin in rat brown adipose tissue of the euthyroid control (black) and hypothyroid groups (grey) treated with methimazole for 7 (M7), 15 (M15), and 21 (M21) days, respectively (**A**). The protein content is expressed as a percentage of the control. Band images from a representative blot of three trials are shown (**B**). Bars represent the mean ± SEM. * Compared to control, * *p* < 0.05.

Further analysis revealed that many peroxisomes often made contact with both mitochondria and sER membranes, forming organellar triads (Figure 4A6–10,B9–12). Triads were present in both euthyroid control and in all experimental groups. Triad number increased over the course of hypothyroidism. This was most apparent on day 21, consistent with an increase in calnexin, a marker of ER enlargement (Figure 5). These findings provide evidence for the hybrid nature of newly born peroxisomes—from the sER and mitochondria.

In addition, peroxisomes localized close to the mitochondrial membrane were observed. Their membrane was in continuity with the outer mitochondrial membrane (Figure 4A11–14,B13–16). This phenomenon was observed in euthyroid control and all experimental groups but was not so frequent. Therefore, we observed peroxisomes as single membrane-bound mitochondria-derived vesicles, which indicated that peroxisomes, in both physiological euthyroid and hypothyroid conditions, could arise from mitochondria.

**Figure 6:**
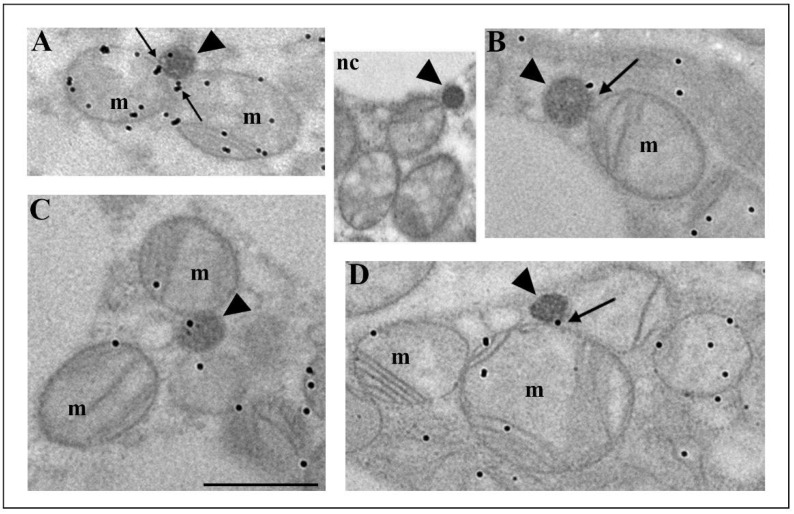
Immunogold labelling of VDAC1, a protein of the mitochondrial outer membrane, in brown adipocytes from both euthyroid and hypothyroid groups. The immunopositive reaction was localized on the mitochondrial outer membrane, on mitochondrial cristae, and on the membrane of some peroxisomes (**A**–**D**, arrowheads). Note the immunopositive reaction on the contact sites of peroxisomes and mitochondria (**A**,**D**—arrows). In addition, we observed peroxisomes that arose by budding from the mitochondrial outer membrane, which was VDAC1 positive (**B**–arrow). Inset nc, negative control of immunolabeling. Original magnification 17,000×, scale bar 1 µm.

To prove the mitochondrial origin of peroxisomes, we employed immunogold labelling of voltage-dependent anion channel 1 (VDAC1), a protein of the outer mitochondrial membrane (Figure 6). Our results showed that VDAC1 was localized on the mitochondrial outer membrane, on mitochondrial cristae, and on the membrane of some peroxisomes (Figure 6A–D). VDAC1-positive peroxisomes were found in the vicinity of mitochondria or in contact with them. In addition, we noticed VDAC1 presence at the mitochondria and peroxisomes contact sites (Figure 6A,D, arrows), indicating the existence of an additional means of communication common to mitochondria and peroxisomes in brown adipocytes. In particular, we observed peroxisomes that arose by budding from the mitochondrial outer membrane (Figure 6B). These peroxisomes may stay in contact with the mother mitochondria for easier communication.

### 3.3. How Extensive Are the Contributions of ER, Mitochondria, or Hybrid Triads to the Observed Biogenesis Pathway, and Are They Affected over the Time Course of Hypothyroidism?

**Figure 7:**
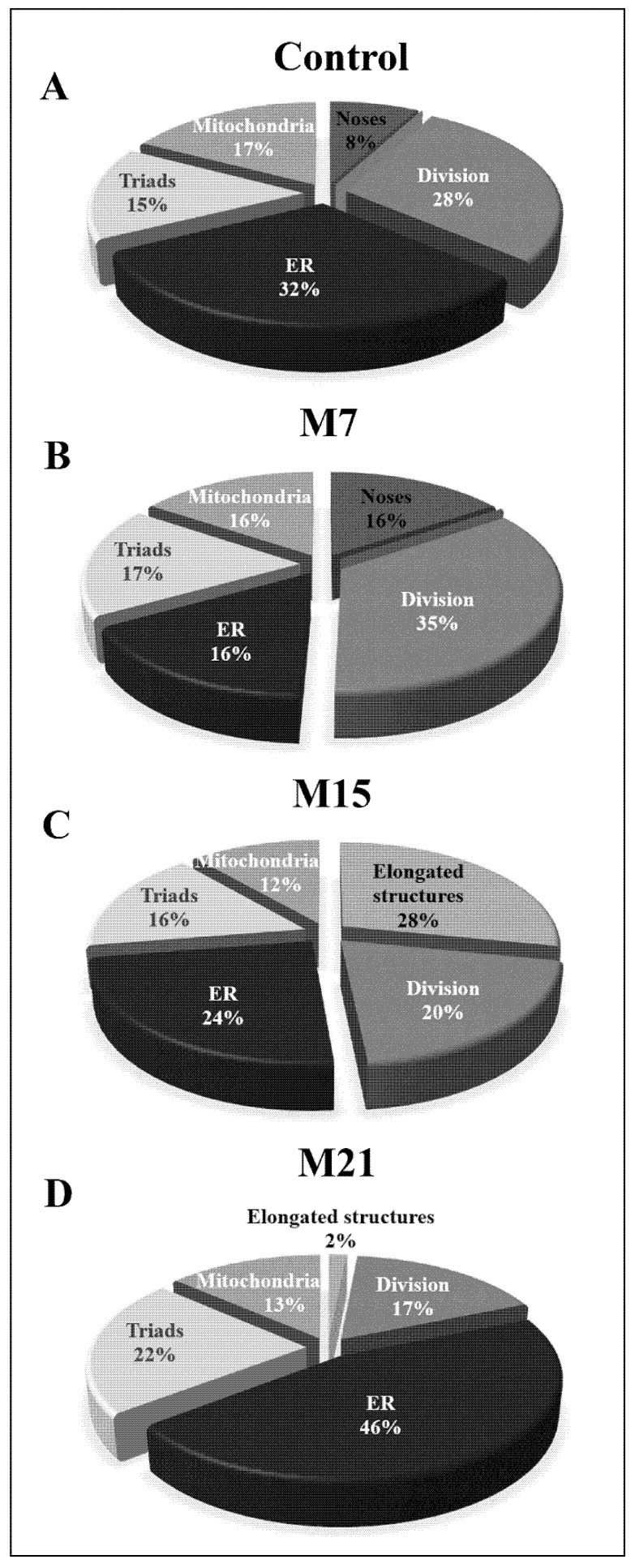
Extent of peroxisomal biogenesis pathways in brown adipocytes (forty cells per group) from euthyroid control (**A**) and hypothyroid groups treated with methimazole for 7 (M7) (**B**), 15 (M15) (**C**), and 21 (M21) (**D**) days, respectively. The relative number of peroxisomal noses (Noses), peroxisomal division present through dumbbell-like structures (Division), peroxisomal tubules (Elongated structures), peroxisomal biogenesis by budding from sER (ER), hybrid nature of peroxisomal origin (Triads), and peroxisomal biogenesis by budding from the mitochondrial outer membrane (Mitochondria) are represented as percentages.

To determine to what extent each of the above-mentioned peroxisomal biogenesis pathways is present in brown adipocytes and to understand how hypothyroidism affects their distribution over time, we analyzed each of them individually in detail. Our results showed that both canonic and *de novo* biogenesis pathways were present in euthyroid and all hypothyroid groups, but to a different extent (Figure 7). In euthyroid control (Figure 7A), the canonic pathway prevailed, and that pattern continued until day 15 when the elongated structures replaced noses (Figure 7C). At day 21, *de novo* peroxisomal biogenesis by budding from the ER became the main pathway (Figure 7D), even though a minor extent of the canonic pathway is still present. The number of triads (hybrid nature of peroxisomal origin) was also highest after 21 days, while biogenesis by budding from the outer mitochondrial membrane stayed at a similar level in euthyroid and all hypothyroid groups.

### 3.4. The Peroxisomal Maturation Level

**Figure 8:**
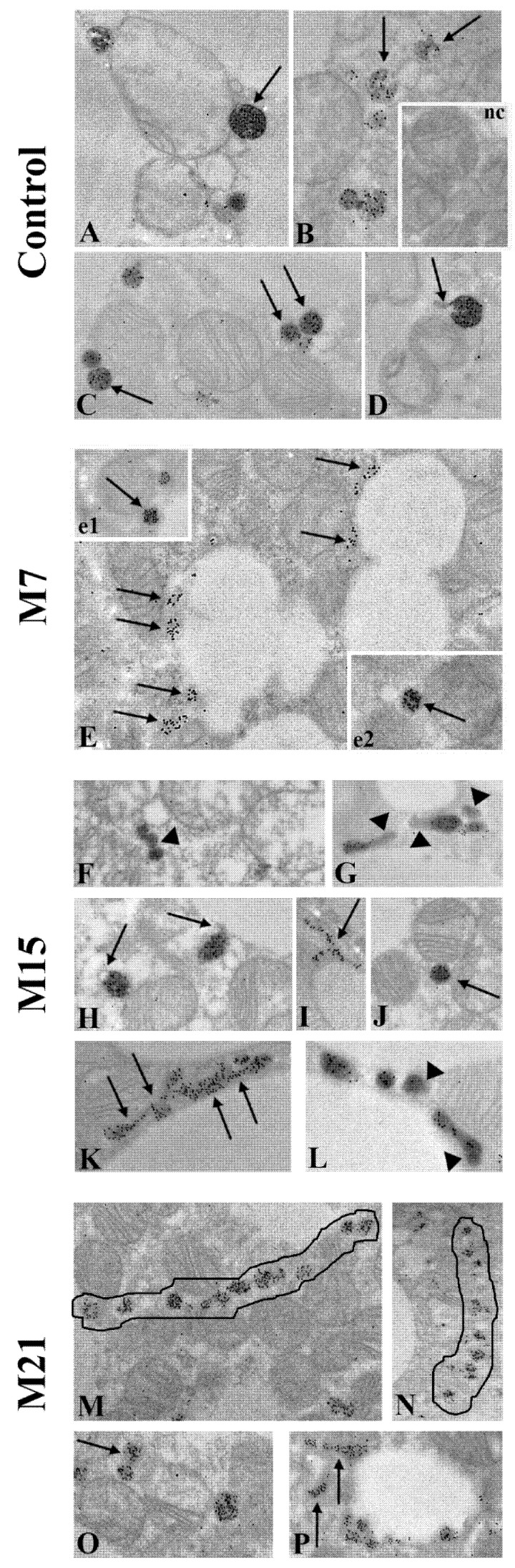
Maturation level of newly born peroxisomes in rat brown adipocytes in euthyroid control (**A**–**D**) and hypothyroid groups treated with methimazole for 7 (M7; **E**,e1,e2), 15 (M15; **F**–**L**), and 21 (M21; **M**–**P**) days, respectively. The strongest immunopositivity for CAT was found in globular, single, fully mature peroxisomes (**A**,**C**,**J**,e1,e2—arrows), but also in peroxisomes, which had started the division process and has a nose (**D**,**H**—arrows). CAT immunopositivity in peroxisomes that originated from the sER (**B**,**I**,**K**,**O**,**P**—arrows) was weaker. Even lower CAT immunopositivity was noticed in peroxisomes within “pearls on strings” which originate from the pre-peroxisomal tubules on day 21 (**M**,**N**—outlined structure). The weakest CAT immunopositivity occurred in peroxisomes close to the lipid bodies on day 7 (**E**—arrows); some peroxisomes within “pearls on strings” (**F**—arrowhead); and in very elongated peroxisomes, which originated from the pre-peroxisomal sER, which were CAT negative in their final narrower part (**G**,**L**—arrowhead). Inset nc, negative control of immunolabeling. Magnification 17,000×.

The degree of peroxisomal maturity is directly proportional to the degree of their functionality. The main marker of peroxisomal maturity is CAT. Using immunofluorescent CAT labelling (Figure 1A), we showed that immunopositivity to CAT was the strongest on day 21 (Figure 1A7). Additionally, immunogold CAT labelling revealed that CAT was localized in the peroxisomal matrix, on their membrane, and in the cytoplasm (Figure 8, but also Figure 2 and Figure 4).

The degree of CAT immunopositivity in peroxisomes depended on the experimental group, as well as on the way of peroxisomal biogenesis. The strongest CAT immunopositivity was noticed in globular, single, fully mature peroxisomes (Figure 8A,C,J,e1,e2). Peroxisomes that arose by the canonic way were smaller than the pre-existing one, poorly labelled by DAB, and less positive to CAT (Figure 2A4–6,B11). Peroxisomes that arose from the sER (Figure 8B,I,K,O,P) also exhibited less CAT staining. The weakest degree of immunopositivity for CAT was observed in very elongated peroxisomes, which originated from the pre-peroxisomal sER, which were CAT negative in their final narrower part (Figure 8G,L and Figure 4B2). In addition, the weakest degree of immunopositivity for CAT was observed in peroxisomes within “pearls on strings”, which originated from the pre-peroxisomal tubules on day 21 (Figure 8M,N). Therefore, our results indicated that in brown adipocytes, there were several populations of peroxisomes, differing according to their way of biogenesis, the rate of their maturation, and perhaps their different functions.

### 3.5. Peroxisomal Biogenesis in Brown Adipocytes in Hypothyroidism Is Driven by a Specific Set of Peroxins and PMP70

**Figure 9:**
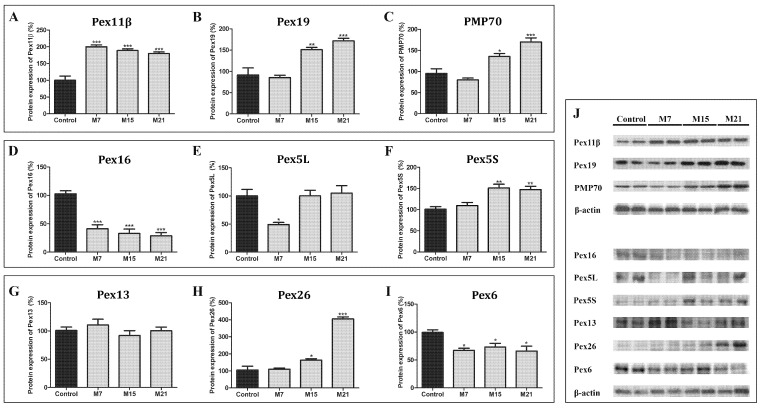
Protein expression of peroxins and PMP70 in rat brown adipose tissue of the euthyroid control (black) and hypothyroid groups (grey) treated with methimazole for 7 (M7), 15 (M15), and 21 (M21) days, respectively. Pex11β (**A**), Pex19 (**B**), PMP70 (**C**), PEX16 (**D**), Pex5L (**E**), Pex5S (**F**), Pex13 (**G**), Pex26 (**H**), Pex6 (**I**). The protein content is expressed as a percentage of the control. Band images from a representative blot of three trials are shown (**J**). Bars represent the mean ± SEM. * Compared to control, * *p* < 0.05, ** *p* < 0.01, *** *p* < 0.001.

Hypothyroidism leads to increased protein expression of some peroxins, while others are decreased (Figure 9). Only Pex13 was not affected. Pex11β protein expression increased on day 7, while Pex19, PMP70, Pex5S, and Pex26 protein expression were increased from day 15 and remained high until the end of treatment, compared to the euthyroid control (Figure 9). The expression of both Pex16 and Pex6 decreased from day 7 until the end of treatment. Pex5L protein expression decreased on day 7 but returned to the control level on both days 15 and 21.

### 3.6. Transcription Regulation of Peroxisomal Biogenesis in Brown Adipocytes

**Figure 10:**
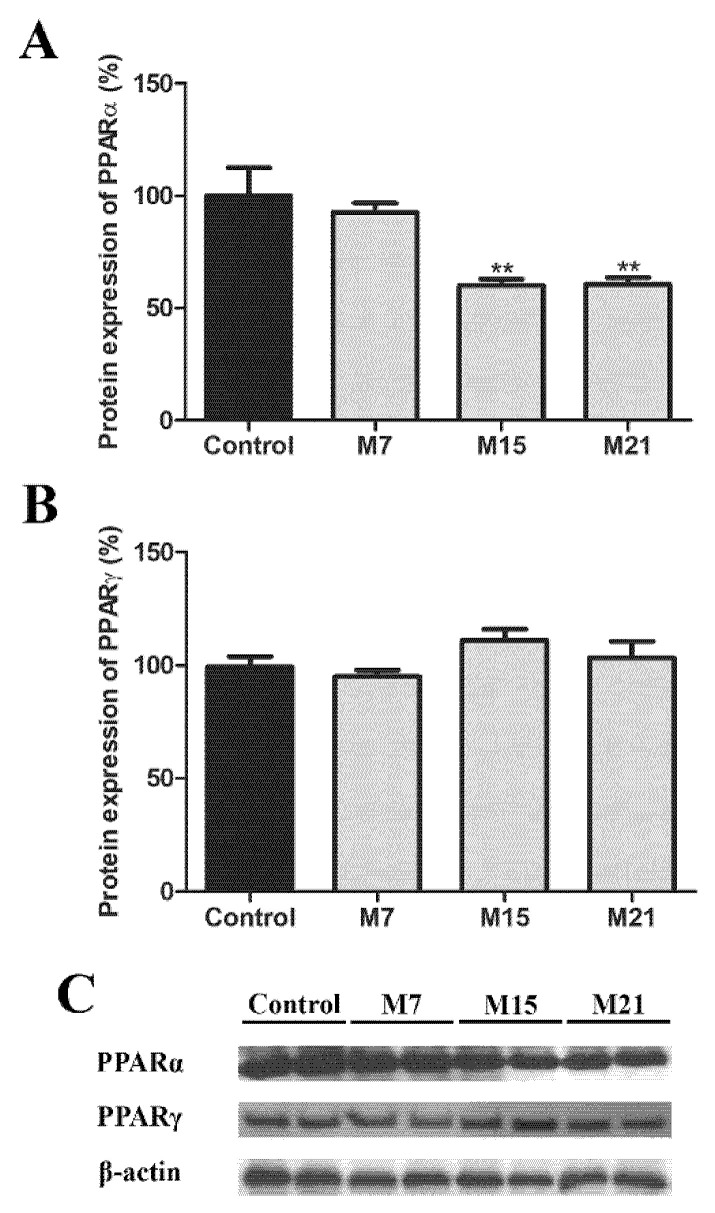
Protein expression of PPARα (**A**) and PPARγ (**B**) in rat brown adipose tissue of the euthyroid control (black) and hypothyroid groups (grey) treated with methimazole for 7 (M7), 15 (M15), and 21 (M21) days, respectively. The protein content is expressed as a percentage of the control. Band images from a representative blot of three trials are shown (**C**). Bars represent the mean ± SEM. * Compared to control, ** *p* < 0.01.

To conclude our study, we looked at the level of transcriptional regulation and analyzed the protein expression of the major transcription factors responsible for peroxisomal biogenesis, PPARα and PPARγ. The protein expression of PPARα, in comparison to the euthyroid group, decreased on both days 15 and 21 (Figure 10A). In BAT, PPARγ is also involved in the transcriptional control of peroxisomal proliferation. Therefore, we analyzed PPARγ protein expression. It remained stable over the time course of hypothyroidism (Figure 10B).

## 4. Discussion

Peroxisomes are organelles characterized by their high plasticity, a reflection of their ability to rapidly adapt their number, shape, size, and enzyme composition according to the cell’s metabolic requirements. Until now, in brown adipocytes, at the ultrastructural level, peroxisomes had been investigated in only two studies [10,11], but their origin still remains uncertain. Herein, we used hypothyroidism induced by methimazole for 7, 15, and 21 days to study peroxisomal remodeling and origin in brown adipocytes. Our results demonstrated that hypothyroidism induced peroxisomal proliferation (increased number by both canonic and *de novo* pathways). Our detailed ultrastructural study revealed for the first time different ways of peroxisomal biogenesis in brown adipocytes: (1) Biogenesis from pre-existing organelles by growth and division, (2) biogenesis from sER by budding, (3) biogenesis from sER and mitochondria—hybrid nature of newly born peroxisomes, and (4) biogenesis from mitochondria by budding from the outer mitochondrial membrane (mitochondria-derived vesicles—MDV). Each pathway took place in the euthyroid control and over the course of hypothyroidism in a time-dependent manner and characterized by specific organellar profiles and sets of peroxins.

### 4.1. Peroxisomal Biogenesis by Growth and Division in Brown Adipocytes

Peroxisomal biogenesis from pre-existing organelles by growth and division is well studied and accepted [19]. Our results demonstrated the existence of this pathway in euthyroid brown adipocytes, which over the time course of hypothyroidism became the major way of peroxisomal biogenesis on day 7 and, to a lesser extent, on day 15. It was a multistep process that required the participation of specific peroxisomal membrane proteins. The peroxin 11 (Pex11) family contains several conserved membrane proteins that control peroxisomal proliferation and regulate peroxisomal morphology, size, and number [20,21,22,23,24,25]. Among them, Pex11β, the constitutively expressed Pex11 isoform, promotes peroxisomal elongation, constriction, and fission, which makes it the main peroxin responsible for this particular pathway of peroxisomal biogenesis [26]. We found that Pex11β was the leading molecular player in peroxisomal biogenesis, highly expressed in hypothyroidism from day 7. At the same time, all the observed structures specific to peroxisomal biogenesis (peroxisomal tubular extension—noses, dumbbell-like, and pearls-on-strings structures) were the most numerous and prevailing pre-peroxisomal structures on day 7, when compared to days 15 and 21. However, high expression of Pex11β until the end of the experiment suggested its role in some other process, perhaps peroxisomal maturation, as postulated by Delille and co-workers [27]. In support of this observation, we found peroxisomal heterogeneity in terms of DAB staining, CAT immunopositivity, and peroxisomal size within “pearls on strings”.

To confirm this, we looked at another important player involved in driving peroxisomal division processes—Drp1. Namely, it has been shown that Drp1 protein is necessary for the final fission of the peroxisomal membrane, but not for elongation and constriction steps that precede it [28]. Our results demonstrated that Drp1 protein level and its presence on peroxisomes was highest on day 7 in comparison to euthyroid control and other hypothyroid groups, validating peroxisomal division as the first step in peroxisomal proliferation. The fall in Drp1 protein expression back to the control level was consistent with reduced peroxisomal division rate on day 15 and especially on day 21. Taken together, our results showed that the growth and division way of peroxisomal biogenesis was an early/first step in peroxisomal proliferation intensified in hypothyroidism in order to produce a large number of new peroxisomes in a short period of time.

### 4.2. Peroxisomal De Novo Biogenesis in Brown Adipocytes

In contrast to peroxisomal biogenesis by the growth and division model, *de novo* biogenesis has been a thorny issue for some time, especially with respect to peroxisomal ER origin. The origin of peroxisomes from both ER and by growth and division in the same cell under physiological conditions is also a subject of contention. Two recent studies arrived at contradictory conclusions that peroxisomes were derived *de novo* and that they were derived exclusively from pre-existing peroxisomes in wild-type yeast, even though both studies measured yeast grown under similar conditions [29,30]. There are similar contradictions about peroxisomal biogenesis in mammals, which have been found to form *de novo* in cells with pre-existing peroxisomes [1].

Kim and colleagues demonstrated that peroxisomes could arise from the ER in mammalian cells and that this was the dominant pathway of peroxisomal formation in both normal and peroxisome depleted mutant cells [31]. Our results correlate with those obtained by Kim and colleagues as we showed peroxisomal biogenesis from the sER in the euthyroid group and we conclude that the sER plays a central role in both *de novo* origin and maintenance of peroxisomes in the cell under euthyroid conditions. Additionally, in a hypothyroid state, *de novo* formation was the dominant way of peroxisomal biogenesis at day 21 of treatment. Accordingly, we can conclude that hypothyroidism induces a two-step increase in the number of peroxisomes. In the first step, on day 7, Pex11β and Drp1 stimulate fast peroxisomal proliferation by growth and division, which continues, but gradually diminishes by day 15. It enhances the *de novo* pathway in the second step by day 21.

Namely, in our study, we found a frequent association of peroxisomes with sER tubules in both euthyroid control and hypothyroidism, with the highest number on day 21. The presence of membranous bridges connecting the peroxisomal with the sER membrane indicates hypothyroidism-induced *de novo* peroxisomal biogenesis from the sER. This is in line with already published data showing cluster-like peroxisomes surrounded by sER and occasionally interconnected in a morphologically defined peroxisomal reticular structure [32]. Later on, the presence of the ER domain forming pre-peroxisomal vesicles whose fusion results in import-competent peroxisomes was confirmed [30,33,34,35] and the presence of Pex13 and PMP70 in the peroxisomal reticulum connected to sER was shown [36], confirming its active role in peroxisomal proliferation. Therefore, besides PMP70 and Pex13, we analyzed peroxins involved in peroxisomal membrane protein import and *de novo* peroxisomal biogenesis (Pex16 and Pex19), and peroxisomal structural/functional maturation (Pex16, Pex19, Pex5, Pex13, Pex26, Pex6).

Regarding the import of peroxisomal membrane proteins (PMPs) and peroxisomal matrix soluble enzymes, data in the literature reveals various underlying molecular mechanisms [37,38]. Pex19 is a predominantly cytoplasmic chaperone and import receptor, but it is also localized to a lesser extent in the peroxisomal membrane [39,40]. It is a multifunctional protein that recognizes peroxisomal membrane target sequences (mPTS) of PMPs; prevents PMPs misfolding, aggregation, and destruction in the cytoplasm; directs newly synthesized PMPs to the peroxisomal membrane, and therefore plays an essential role in PMPs import [40]. Here we found that hypothyroidism increased Pex19 protein expression from day 15 to the end of treatment, which demonstrated its importance in brown adipocyte peroxisomal biogenesis. In that way, Pex19 expression was coupled to the synthesis of new PMPs and the increased need for their stabilization and insertion into newly formed pre-peroxisomes.

The peroxisomal membrane protein that helps Pex19 by enabling the insertion of newly formed PMPs is Pex16. Being an integral membrane protein in the ER and peroxisomes, Pex16 is considered to be a master regulator of *de novo* peroxisomal biogenesis from the sER [8,31]. Surprisingly, hypothyroidism decreased the protein expression of Pex16 on day 7 and it remained low until the end of the experiment. One possible explanation is that *de novo* biogenesis demands, at least in brown adipocytes, very rapid peroxisomal maturation along with their formation, enabling the newly born peroxisomes to immediately become fully functional. Given that depletion of endogenous Pex16 itself increased the direct incorporation of PMPs [5], our results indicate that hypothyroidism probably favors direct incorporation of PMPs to peroxisomes via Pex19, which hinders Pex16 expression. This is supported by the fact that hypothyroidism induced PMP70 protein expression in parallel with Pex19 from day 15 until the end of treatment. Thus, increasing expression of PMP70 implies the existence of large numbers of functionally mature peroxisomes, since PMP70 serves to transport fatty acids from the cytosol into peroxisomes, for oxidation [41].

Peroxisomal matrix proteins are targeted to the peroxisomes via one of two peroxisomal targeting signals (PTS1 and PTS2) [42], bound by cycling cytosolic receptors Pex5 and Pex7, respectively [43]. Pex5 exists in two isoforms, a long, Pex5(L), and a shorter form, Pex5(S). Since Pex5(L) contains a Pex7 binding epitope and acts as a Pex7 coreceptor for PTS2 proteins, the two import pathways converge at its level [44]. Our results demonstrated that Pex5(L) protein expression was reduced on day 7 of hypothyroidism but then it returned to the control level by day 15. In contrast, Pex5(S) protein expression was stable at day 7 but increased on day 15 of hypothyroidism and remained increased. Hence, our results suggest that early hypothyroidism favors import of PTS1 proteins, but since they are more numerous than PTS2 this trend continues until the end of the treatment, through Pex5(S) activity. Export of Pex5 from peroxisomes is driven by the Pex1/Pex6 complex, anchored in the membrane by direct binding of Pex6 to Pex26 [45,46,47]. Weller and co-workers showed that Pex26 deficiency impaired peroxisomal import of both PTS1- and PTS2-targeted matrix proteins in skin fibroblast cell lines [48]. It is interesting that import of Pex26 depends on Pex19 [49] and that Pex26 follows the Pex19- and Pex3-mediated direct import pathway [50]. We found that the Pex26 protein expression level started to increase from day 15 and remained high on day 21 of hypothyroidism in comparison to euthyroid control. This was a similar pattern to that observed for Pex19. Conversely, hypothyroidism induced a strong decrease in Pex6 protein expression, which did not affect catalase import, as we demonstrated by CAT immunogold labelling.

Another intriguing peroxisomal origin has been shown to exist. Namely, there is growing evidence that mitochondria are also involved in peroxisomal biogenesis. In line with that, we observed that many peroxisomes were often in contact with mitochondrial and sER membranes as a triad of organelles. The number of triads increased over the time course of hypothyroidism, the most numerous observed on day 21. Because of the clear presence of membranous bridges between peroxisomes and mitochondria on one side and ER on the other, such findings provide evidence of the hybrid nature of newly born peroxisomes—from sER and mitochondria. More than forty years ago, Goldfisher and co-workers discovered a relationship between the absence of peroxisomes and altered mitochondrial morphology in patients with cerebro-hepato-renal syndrome [51]. Many years later, Neuspiel and colleagues first reported vesicular transport and communication between mitochondria and peroxisomes by mitochondria-derived vesicles [6]. Based on this evidence and others, Mohanty and McBride proposed that both ER and mitochondrial-derived vesicles could contribute to peroxisomal origin [7]. Moreover, Sugiura and co-workers provided evidence of the hybrid nature of newly born peroxisomes within human fibroblast cell lines from patients with Zellweger syndrome, lacking peroxisomes [8]. This hybrid nature of peroxisomal biogenesis includes forming pre-peroxisomal vesicles from the mitochondrial outer membrane, carrying Pex3 and Pex14, whose maturation needs fusion with ER-derived vesicles, carrying Pex16, to form fully import-competent pre-peroxisomes. Herein, we did not observe any vesicles between the mitochondria and sER, but we noticed mature peroxisomes, CAT positive, with membranous bridges, which were connected to the mitochondrial outer membrane and sER membrane. We assume that these peroxisomes originate by fusion of pre-peroxisomal vesicles derived from the outer mitochondrial membrane and sER membrane, respectively, but these vesicles stay in contact with parental organelles during maturation events. We propose that this subpopulation of peroxisomes in brown adipocytes stay in a morpho-functional relationship with organelles from which they originate because of easier metabolic crosstalk within triads, and control of lipid metabolism in methimazole-induced hypothyroidism.

In our study, we noticed peroxisomes as single membrane-bound mitochondria-derived vesicles, whose membrane was continuous with the outer mitochondrial membrane. These vesicles were observed in euthyroid and all hypothyroid groups but were a rare occurrence. These single membrane-bound vesicles looked like mature peroxisomes, were CAT positive, and for the first time, indicated the appearance of peroxisomes derived only from mitochondria. Hence, this phenomenon indicated that peroxisomes, in both physiological and hypothyroid states, could arise by budding from mitochondria. Further evidence that supports this indication was the presence of VDAC1 at the peroxisomal membrane of brown adipocytes. Up to now, VDAC1 has been identified in peroxisomes of yeast and oilseed glyoxysomes [52], but not in peroxisomes of mammalian cells [53]. Our results, for the first time, showed peroxisomal localization of VDAC1. Hence, common expression of VDAC1 for mitochondria and peroxisomes can provide additional evidence that some peroxisomes originate from mitochondria. Furthermore, the presence of VDAC1 at the contact sites between mitochondria and peroxisomes indicated an additional mode of inter-organellar communication and perhaps metabolite transport. Therefore, we propose that there are more populations of peroxisomes in brown adipocytes, which differ both in the manner of their origin and their function. This may not be surprising since the presence of a heterogeneous peroxisomal population has already been shown in rat liver [54], with different locations in the cytoplasm and composition with other organelles, with different enzyme content and consequently different function [55,56].

Clearly, hypothyroidism increased all ways (within canonic and *de novo* pathways) of peroxisomal biogenesis in brown adipocytes, but origin from pre-existing organelles, by growth and division, and by budding from the sER prevailed. However, not all different ways of peroxisomal biogenesis simultaneously operate in a single cell. Triads of organelles, peroxisome−sER−mitochondria were present in all experimental groups but were the most numerous on day 21. For the first time, we observed peroxisomal biogenesis by mitochondria-derived vesicles (MDV) in both the euthyroid control and hypothyroidism, indicating that mitochondria play an important role in both *de novo* origin and maintenance of peroxisomes, at least in brown adipocytes. It also points out peroxisomal significance in brown adipocytes and their participation as a potential “equal partner” to mitochondria in hypothyroidism. Specific peroxisome-mitochondria communication can be achieved directly through physical contact sites or indirectly via vesicular transport or signaling molecules such as lipids, metabolites, or reactive oxygen species [57,58]. Our results demonstrated that in brown adipocytes, MDV only play a role in peroxisomal biogenesis. Vesicular transport, however, cannot be excluded.

### 4.3. Peroxisomal Maturation Level

Peroxisomal formation is followed by maturation, which is accompanied by the selective and gradual import of peroxisomal membrane proteins and lipids, as well as matrix proteins [59]. The degree of peroxisomal maturity is directly proportional to the degree of their functionality. The main marker of peroxisomal maturity is CAT. Using CAT immunogold labelling, we confirmed that peroxisomes originated via different biogenesis pathways and were differentially matured. Namely, we found that peroxisomes with noses were CAT positive only in the main globular part. In addition, many tubular structures, which appeared from day 15 of treatment, were CAT positive only in their initial wider part. Peroxisomes whose membranes were connected to the membrane of sER, exhibited homogeneous CAT positivity in their matrix. In addition, peroxisomes within triads were CAT positive only in their globular part, and peroxisomes that arose by budding from mitochondria were also CAT positive. The absence of CAT in peroxisomal noses, the final narrower part of tubular structures, and in the area of the bridges that connect peroxisomes with ER, triads, and mitochondria, confirms that CAT can be imported only into fully mature peroxisomes [8]. Interestingly, peroxisomes with a CAT negative tail were first described as a gastruloid cistern, or CAT negative membrane loop [60,61]. A decrease in CAT activity was suggested by a reduction of the electron density of the peroxisomal matrix, frequently observed in hepatitis, and by a heterogeneous distribution of the CAT reaction product leaving a small transparent zone [62]. In contrast, CAT positive peroxisomes whose membrane is continuous with the outer mitochondrial membrane, a membrane of sER, or both within triads, provides strong evidence for peroxisomal *de novo* biogenesis in rat brown adipocytes.

The degree of peroxisomal CAT immunogold positivity increases over the time course of hypothyroidism and largely depended on the method of peroxisomal biogenesis. Accordingly, we recognized three peroxisomal populations in brown adipocytes: Strong CAT positive mature peroxisomes (single, globular), less CAT positive immature peroxisomes (“pearls on a string”), and rare CAT negative peroxisomal profiles (on day 15 only). Hence, peroxisomal abundance in brown adipocytes in hypothyroidism is heterogeneous in terms of maturity degree. This is in line with previous reports regarding the time and manner of peroxisomal maturation [27]. We did not find differences in immature peroxisomes. No data in mammalian cells from other research groups exist. Interestingly, five different immature peroxisomal populations maturing at ordered pathway have been identified in yeast *Yarrowia lipolytica* [63].

We also observed division only in mature peroxisomes in both the euthyroid control and hypothyroidism. However, there is no consensus in the literature regarding the division of mature and immature peroxisomes in both yeast and mammalian cells. In *Yarrowia lipolytica* and *Hansenula polymorpha*, peroxisomal vesicles divide only after acquiring maturity [63,64], while in *Candida boidinii*, immature peroxisomes with partial import of matrix proteins are prone to division [65]. In human cells, however, both mature and immature peroxisomes can divide [38].

### 4.4. Transcriptional Regulation of Peroxisomal Biogenesis

Peroxisomal biogenesis is transcriptionally regulated by peroxisome proliferator-activated receptors (PPARs), ligand-activated transcription factors that belong to a subfamily of nuclear receptors. Three of the most prominent members, PPARα, PPARγ, and PPARδ (β) [66], show distinct tissue distribution patterns and target gene expression profiles. PPARα is highly expressed in liver and brown adipose tissue, where it is a key regulator of fatty acid oxidation. PPARδ is ubiquitously expressed, involved in adipocyte development, and exerts significant functional overlap with PPARα. PPARγ is highly expressed in adipose tissue and is required for adipogenesis and overall BAT metabolism [2,9,67]. PPARα was first linked to peroxisomal proliferation; its activation not only increases the expression of fatty acid oxidation genes but also the abundance of peroxisomes in the liver [67]. In contrast, it was shown that fibrates, which are highly effective in inducing peroxisomal gene activation via PPARα in the liver [68], do not regulate the expression of peroxisomal genes in BAT [69].

Importantly, there is evidence that in brown adipose tissue, peroxisomal proliferation correlates with the expression of PPARα and PPARγ [9]. Moreover, recent data showed that PPARα/γ shared target genes engaged in the regulation of the thermogenic function of brown adipocytes [70]. In such a setting, our results suggest that in hypothyroidism, the regulation of peroxisomal biogenesis in brown adipocytes may be a result of mutual action of both PPARα and PPARγ. PPARγ expression, as a master regulator of brown adipocytes’ structure and function, is not affected, while the expression of Pex 16, peroxin, which is a direct and functional PPARγ target gene [71], is strongly affected by hypothyroidism. Decreased PPARα expression on days 15 and 21 along with Pex16 strong downregulation could serve to inhibit peroxisomal overproduction. Finally, PPARα/γ are probably not the only/necessary mechanism for the modulation of the expression of the main molecular players at the core of peroxisomal biogenesis.

## 5. Conclusions

In summary, we have described that hypothyroidism induces peroxisomal proliferation in brown adipocytes by exploiting different peroxisomal biogenesis ways. Peroxisomes originate from pre-existing organelles by growth and division, from sER by budding, or sER and mitochondria as hybrid in nature, and from mitochondria. Each pathway operates in the euthyroid control and during the course of hypothyroidism in a time-dependent manner and is characterized by specific organellar profiles and peroxins. Peroxisomal presence, distribution, and degree of maturation are heterogeneous in brown adipocytes in a Harlequin-like manner and reflect the differences in peroxisomal origin. Given the relevance of multiple organellar contacts required for thermogenesis in brown adipocytes, clarification of the structural basis of their associations clearly beckons future research.

## Data Availability

Not applicable.

## Supplementary Materials

The following are available online at https://www.mdpi.com/article/10.3390/cells10092248/s1, Supplementary Material_1_Serum levels of T3, T4, and TSH. Supplementary Material_2_Video-Supp_1-4.AVI_Immunofluorescent labelling of catalase.
