# Supplementary material for: Hypothyroidism Intensifies Both Canonic and the De Novo Pathway of Peroxisomal Biogenesis in Rat Brown Adipocytes in a Time-Dependent Manner
Source: Cells. 2021 Aug 30;10(9):2248. doi: 10.3390/cells10092248 (PMC8472630; doi:10.3390/cells10092248)

Supplementary Material 1

Serum levels of T3, T4, and TSH

Serum levels of triiodothyronine (T3), thyroxine (T4) and thyroid-stimulating hormone (TSH) were determined by RIA method (T3, T4) (INEP, Serbia) or by commercial ELISA kit (TSH) (CSB E05115r, Cusabio, USA) from six animals per group. Compared to euthyroid control (black), in hypothyroid groups (grey) treated with methimazole for 7 (M7), 15 (M15), and 21 (M21) days respectively, serum levels of T3 and T4 decreased, while the level of TSH increased over the course of hypothyroidism. Bars represent the mean ± SEM. *Compared to control, *p<0.05, **p<0.01, ***p<0.001.

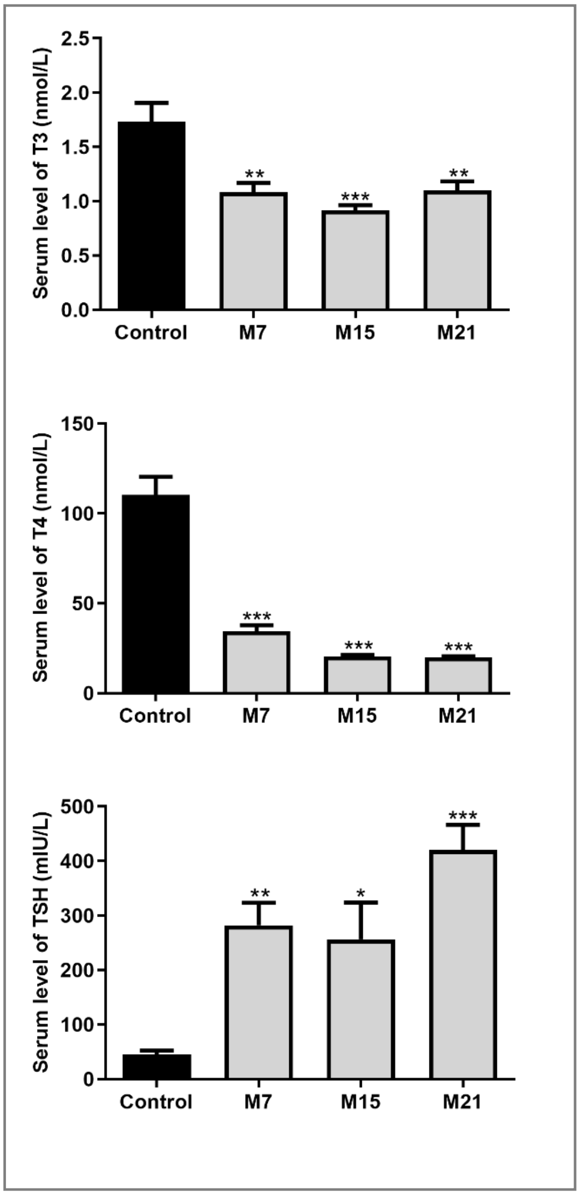

Supplement: Supplementary file 1 [file cells-10-02248-s001.zip › Supplementary Material 1_Serum levels of T3, T4, and TSH.pdf]
