# Supplementary material for: Hypothyroidism Intensifies Both Canonic and the De Novo Pathway of Peroxisomal Biogenesis in Rat Brown Adipocytes in a Time-Dependent Manner
Source: Cells. 2021 Aug 30;10(9):2248. doi: 10.3390/cells10092248 (PMC8472630; doi:10.3390/cells10092248)
Supplement: Supplementary file 1 [file cells-10-02248-s001.zip › Supplementary Material_2_video.pdf]

Supplementary Material 2

Video data – Immunofluorescent labelling of catalase

Semi-thin sections (2 μm) of interscapular depo of brown adipose tissue were used for standard immunolabeling procedure, using primary antibody against CAT, and appropriate fluorochrome-conjugated secondary antibody (1:400; Alexa Fluor® 488 goat anti-rabbit, Thermo Fisher Scientific, USA). Sytox orange (1 μL ml-1, Thermo Fisher Scientific) was used for nuclei counterstaining. Slides were mounted with Mowiol (Polysciences, Eppelheim, Germany), and confocal images were acquired with a Leica TSC SP8 confocal microscope (Leica Microsystems). Z-stacks of images of 1024 x 1024 pixels were acquired using 63x/1.4 NA oil-immersion objective, with 7x scan zoom to meet the Nyquist criterion. The 3d reconstruction of z-stacks was done using LAS AF (Leica Application Suite, Advanced Fluorescence Lite, 2.6.3) software and exported as video in AVI file format.

CAT – green, Sytox orange – blue, nuclei. Euthyroid control – Control, hypothyroid groups treated with methimazole for 7 (M7), 15 (M15), and 21 (M21) days.
